# Hirudin as a Therapeutic and Brain-Delivery Platform: From Stroke to Central Nervous System Disorders

**DOI:** 10.3390/ijms27146388

**Published:** 2026-07-18

**Authors:** Yufei Sun, Qiang Fu, Kunyao Deng, Hongjie Zhang, Zhong Zuo, Zhonggui He, Zhijun Yang

**Affiliations:** 1 School of Chinese Medicine, Hong Kong Baptist University, Hong Kong SAR, China; 22256245@life.hkbu.edu.hk (Y.S.); zhanghj@hkbu.edu.hk (H.Z.); 2 Wuya College of Innovation, Shenyang Pharmaceutical University, Shenyang 110016, China; fuqiang@syphu.edu.cn (Q.F.); hezhonggui@vip.163.com (Z.H.); 3 School of Pharmaceutical Engineering, Shenyang Pharmaceutical University, Shenyang 110016, China; 15108013787@163.com; 4 School of Pharmacy, The Chinese University of Hong Kong, Shatin, N.T., Hong Kong SAR, China; joanzuo@cuhk.edu.hk

**Keywords:** hirudin, stroke, drug delivery, peptide therapeutics, blood–brain barrier, CNS translational research

## Abstract

Crossing the blood–brain barrier (BBB) remains a central obstacle in central nervous system (CNS) therapeutics. Hirudin, a 65-amino acid, disulfide-stabilized, direct thrombin inhibitor with a long history in Traditional Chinese Medicine, has shown compelling efficacy in thrombotic disorders and mounting neuroprotective activity in preclinical stroke models, with supportive clinical signals. Although few studies have directly quantified its BBB permeability, available evidence indicates low but measurable brain exposure under pathological conditions, likely via paracellular leakage rather than receptor-mediated transport. To extend its therapeutic reach in cerebrovascular diseases and mitigate bleeding risk, researchers have engineered hirudin using nanocarrier encapsulation, hydrogel-based delivery systems, prodrug strategies, and other methods to enhance targeting, prolong half-life, and localize activation. These approaches also enable synergistic combinations with established CNS drugs and may facilitate delivery of co-therapeutics to the brain. This review synthesizes current evidence for hirudin in stroke, neurodegeneration, psychiatric disorders, and brain tumors; evaluates its context-dependent BBB access; and outlines a translational agenda centered on quantitative brain pharmacokinetics, pathology-guided targeting, stimulus-responsive formulations, controlled clinical evaluation, and rigorous management of bleeding risk and chemistry, manufacturing, and controls (CMC) readiness.

## 1. Introduction

The blood–brain barrier (BBB) preserves central nervous system (CNS) homeostasis by tightly regulating the exchange of substances between blood and brain parenchyma [1]. It is formed by specialized endothelial cells sealed by tight junctions, supported by pericytes and astrocytic end-feet, and reinforced by transporters and metabolic enzymes [2]. Most small molecules with molecular weight outside ~400–600 dalton (Da) or with low lipophilicity exhibit poor passive permeation; as a result, over 98% of small-molecule drugs and nearly all biologics (e.g., antibodies, nucleic acids) are excluded from the brain [2,3,4,5]. Although increasing lipophilicity can improve diffusion, it often increases off-target toxicity [4]. Consequently, efficient, safe delivery of anti-stroke, anti-psychotic, and anti-tumor agents to the brain remains difficult.

Certain peptides, by virtue of structure and charge, can traverse or bypass the BBB and have shown promise in CNS disorders [6]. Hirudin is a single-chain, 65-amino acid polypeptide secreted by medicinal leeches, long used in Traditional Chinese Medicine to promote circulation and resolve stasis [7]. Its N-terminal domain mediates potent, direct thrombin inhibition; the hydrophilic C-terminus contributes to solubility; and three disulfide bonds in the cysteine (Cys) sites (Cys6–Cys14, Cys16–Cys28, Cys32–Cys39) stabilize the fold [8,9]. Beyond cardiovascular and cerebrovascular disease, hirudin and its analogs have been studied in cancer, diabetes, and dermatologic conditions [7,10,11]. In stroke, BBB disruption likely facilitates brain entry [12], and multiple studies indicate neuroprotective effects in cerebral ischemia models [13], raising interest in hirudin both as a therapeutic and as a “molecular carrier” to aid brain delivery. This review synthesizes advances in stroke, examines the evidence and putative mechanisms for BBB penetration, and evaluates engineering strategies that position hirudin as a platform for brain delivery in diverse CNS diseases.

## 2. Hirudin for Treating Stroke

Stroke is a leading cause of death and disability worldwide [14]. Approximately 85% are ischemic strokes resulting from thromboembolism or endothelial dysfunction, which interrupt cerebral blood flow and trigger cascades of metabolic failure, inflammation, and cell death [15]. Thrombin is a central effector in thrombosis and ischemia–reperfusion injury: it converts fibrinogen to fibrin, activates protease-activated receptors, and propagates neuroinflammation, edema, and BBB dysfunction [16,17,18]. Elevated thrombin following cerebral ischemia activates microglia and contributes to neuronal and astrocytic injury, exacerbating secondary damage [18,19].

### 2.1. The Anticoagulant Mechanism and Pharmacological Advantages of Hirudin

**Figure 1:**
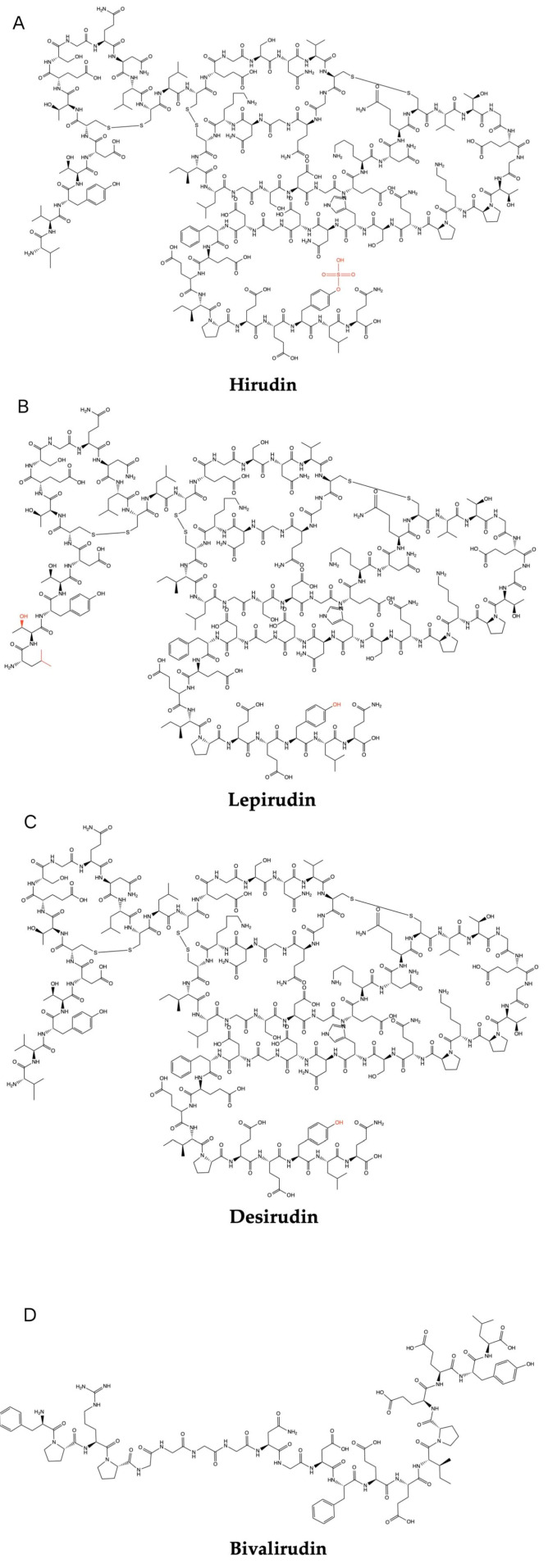
Chemical structures of hirudin, its derivatives, and a synthetic hirudin-based compound. Structural differences between hirudin and its derivatives are noted in red. (**A**) Hirudin. Chemical formula: C_287_H_440_N_80_O_113_S_7_. (**B**) Lepirudin, a recombinant hirudin derivative. Chemical formula: C_287_H_440_N_80_O_111_S_6_. (**C**) Desirudin, (also known as 63-Desulfohirudin), another recombinant hirudin derivative. Chemical formula: C_287_H_440_N_80_O_110_S_6_. (**D**) Bivalirudin, a synthetic 20-amino acid peptide. Chemical formula: C_98_H_138_N_24_O_33_.

**Figure 2:**
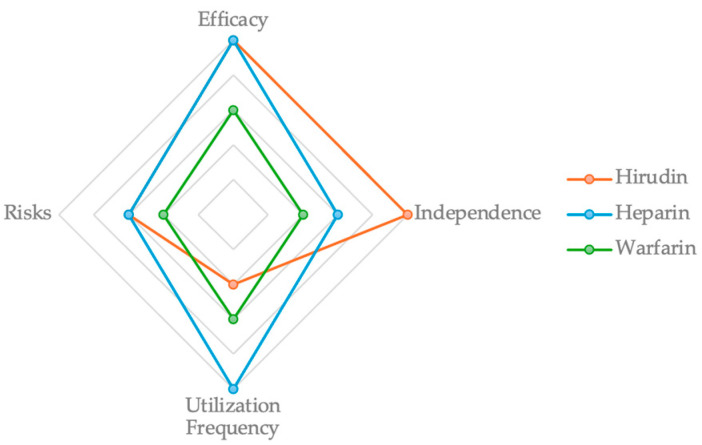
Radar chart comparing hirudin, heparin and warfarin based on their efficacy, independence, risks, and utilization frequency.

Hirudin is a direct thrombin inhibitor with a strong affinity constant ranging from 0.1 to 0.3 pM [20]. It binds thrombin in a 1:1 stoichiometry independent of antithrombin III and inhibits both free and fibrin-bound thrombin [21,22]. Compared with heparin, hirudin avoids heparin-induced thrombocytopenia and platelet interactions [23]. Unlike warfarin, as a vitamin K antagonist that requires frequent international normalized ratio (INR) monitoring, hirudin’s direct, selective thrombin blockade yields more predictable anticoagulation [24]. Several clinically used agents derive from the hirudin pharmacophore: lepirudin and desirudin are recombinant hirudin variants, whereas bivalirudin is a synthetic hirudin-based peptide (Figure 1). Collectively, these agents complement or improve upon heparin and warfarin in specific scenarios (Figure 2).

Multiple in vitro and in vivo studies support hirudin’s antithrombotic efficacy. Thrombosis underlies major arterial (e.g., myocardial infarction, ischemic stroke) and venous (deep vein thrombosis, pulmonary embolism) diseases [25]. Hirudin and its analogs have shown benefit across these settings. For example, biomimetic nanoparticles loaded with hirudin targeted thrombi and improved lipid metabolism and inflammation in atherosclerosis models [26,27]. In myocardial ischemia–reperfusion injury and hemorrhagic shock/resuscitation, hirudin (1 mg/kg) reduced cardiac injury biomarkers (Troponin T, Creatine Kinase-MB), suppressed macrophage M1 polarization and pyroptosis-associated factors, and improved left ventricular function (ejection fraction, fractional shortening) [28]. In acute pulmonary embolism, subcutaneous recombinant hirudin lowered mean pulmonary artery pressure, vascular wall thickening, and apoptotic cell counts, and improved histopathology [26]. The low-molecular-weight RGD-hirudin also prolonged the blood clotting time in vitro and prevented coagulation in the rat thrombosis model [29].

Especially in stroke treatment, hirudin exhibits multi-modal action, including anticoagulation, anti-inflammatory effects, preservation of the BBB, and neuroprotection. Inflammasome activation is a critical driver of ischemic brain injury. In middle cerebral artery occlusion/reperfusion (MCAO/R) rats, hirudin (40 mg/kg) suppressed NOD-like receptor protein 3 (NLRP3) inflammasome components; pharmacological activation with nigericin attenuated this effect [12,28,30]. Hirudin also alleviates edema, stabilizes the BBB, and dampens microglial activation and cytokine release. In subarachnoid hemorrhage (SAH), hirudin reduced brain water content, Evans blue extravasation, and proinflammatory cytokine levels (TNF-α, IL-6, IFN-γ), and improved neurological scores [30]. In MCAO/R and oxygen–glucose deprivation/reperfusion (OGD/R) models, hirudin enhanced microglial viability and supported brain microvascular endothelial cell survival, migration, and tube formation in association with Wnt/β-catenin signaling, reducing infarct volume and neurological deficits in vivo [12,31].

### 2.2. Clinical Studies of Hirudin for Treating Stroke

**Figure 3:**
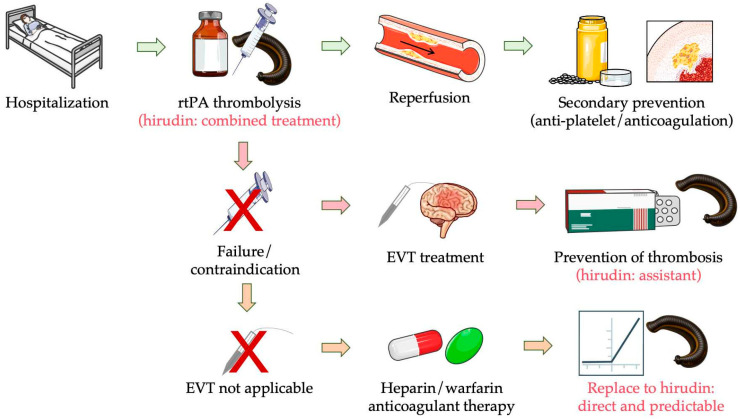
Schematic illustration of current clinical therapies for stroke and the potential for expanded use of hirudin. rtPA: recombinant tissue plasminogen activator. EVT: endovascular therapy. The roles of hirudin are explained and illustrated in pink.

Clinical stroke management aims to prevent events, minimize acute injury, and promote recovery via thrombolysis, endovascular therapy (EVT), antithrombotics, and supportive care [32,33,34,35,36,37]. Hirudin is relevant as a direct thrombin inhibitor in several contexts (Figure 3).

Recombinant tissue plasminogen activator (rtPA) is the standard thrombolytic in acute ischemic stroke but is limited by a narrow time window, incomplete recanalization, hemorrhagic transformation, and neurotoxicity [32]. Adjunct direct thrombin inhibition may help prevent micro-thrombosis and re-occlusion, though dosing and bleeding risk require careful evaluation. Novel delivery concepts (e.g., ultrasound-responsive carriers for controlled rtPA release) show promise in preclinical studies but need clinical validation.

Guidelines recommend anticoagulation for cerebral venous thrombosis, typically with initial low-molecular-weight heparin (LMWH) followed by vitamin K antagonists for 3–12 months or longer if risk persists [35]. Venous hemorrhage does not contraindicate anticoagulation, although full-dose anticoagulants are generally avoided in acute arterial ischemic stroke [36]. Given its pharmacology, hirudin is a plausible alternative in selected situations.

EVT has transformed outcomes for large-vessel occlusions [33,35]. In this setting, peri-procedural hirudin could mitigate procedure-related thrombosis and support sustained reperfusion, but prospective trials are needed.

Trials of hirudin-class agents indicate more predictable anticoagulation than heparin, with variable bleeding risk depending on dose and context. For example, OASIS-2 reported slightly higher major bleeding with hirudin versus heparin (1.2% vs. 0.7%) but fewer composite ischemic events (5.6% vs. 6.7%) [38,39,40]. In secondary prevention of cardioembolic stroke due to atrial fibrillation, a hirudin–aspirin regimen reduced safety events relative to warfarin (HR 0.27) [24]. Ongoing and completed studies of recombinant hirudin variants (e.g., lepirudin, desirudin) and analogs (e.g., bivalirudin) continue to refine their roles; phase IV data in deep vein thrombosis (DVT) prophylaxis may add supportive evidence [41]. Determining optimal dosing that balances efficacy with intracranial bleeding risk and defining cost-effectiveness comparing LMWH remain priorities [42].

## 3. BBB Penetration of Hirudin

### 3.1. The Structure of the BBB

**Figure 4:**
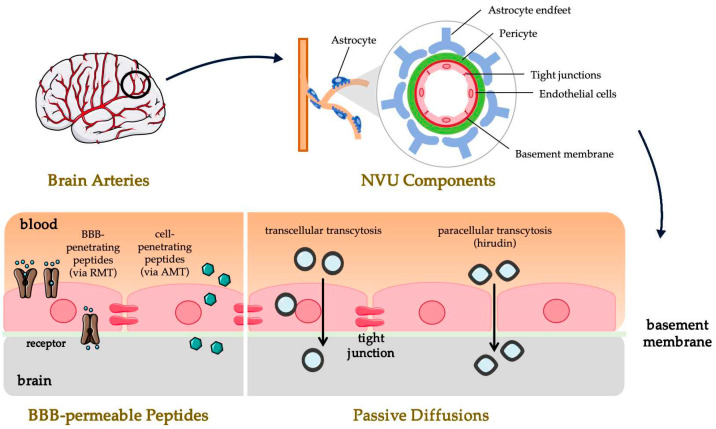
Schematic illustration of the neurovascular unit (NVU), and BBB-permeable peptides and passive diffusion routes of BBB penetration. RMT: receptor-mediated transcytosis. AMT: adsorptive-mediated transcytosis.

The BBB represents a dynamic and highly regulated interface, far beyond a static anatomical structure [5]. It functions as an integrated neurovascular unit (NVU) (shown in Figure 4), in which the coordinated interactions of multiple cell types enforce strict control over substance exchange between the bloodstream and the CNS [2]. This sophisticated biological system indicates a fundamental challenge in neuro-therapeutics, making the quest to safely and efficiently circumvent or cross the BBB a central challenge for drug delivery. It is this very challenge that underpins our study on the potential of hirudin, a large polypeptide, to traverse this barrier. The core anatomical and functional elements of the NVU include:

- Endothelial cells: Cerebral microvascular endothelial cells form the core physical barrier [43]. They are characterized by continuous tight junctions (TJs)—complexes of transmembrane and peripheral proteins that create occluding junctions or zonulae occludes [4,5]. These TJs eliminate fenestrations and minimize non-specific pinocytic activity, effectively blocking the paracellular passage of most hydrophilic molecules, ions, and macromolecules [2,44]. Furthermore, the endothelium actively maintains BBB integrity through a negatively charged glycocalyx that repels anionic molecules, the precise expression of specific influx and efflux transporters, and an exceptionally high trans-endothelial electrical resistance that restricts transcellular vesicular traffic [45,46,47].
- Pericytes: Embedded within the vascular basement membrane, pericytes are integral regulators of the BBB. They are mural cells of blood microvessels, which are essential for vascular development, stability, and homeostasis [48]. Pericytes directly regulate BBB-specific gene expression patterns in endothelial cells, guide the polarization of astrocyte end-feet, and modulate cerebral blood flow and neuroinflammatory responses [5,49].
- Astrocytes: Astrocytes extend specialized “end-feet” that extensively ensheathe the capillaries, forming a crucial link between neurons and the vasculature. They contribute to BBB integrity by releasing soluble factors that promote the barrier phenotype and by dynamically regulating vascular permeability [50,51]. Their end-feet contain specialized organelles and scaffold proteins that anchor a multitude of channels, transporters, and enzymes essential for neurovascular coupling and ionic homeostasis [52]. Disruption of astrocyte-endothelial adhesion is a common feature of various neurological disorders, including stroke and Alzheimer’s disease, leading to barrier impairment [53,54].
- Basement membrane: Secreted collectively by endothelial cells and astrocytes, the basement membrane is a specialized, 50–100 nm extracellular matrix layer [54]. Composed primarily of collagen IV, laminin, nidogen, perlecan, and agrin, it provides critical structural support and an additional filtration barrier [54,55]. Beyond its mechanical role, it is active in signal transduction, and its integrity is crucial [56]; for instance, its dissolution during ischemic stroke is a key event in BBB disruption [55]. We hypothesize that pathological alterations in the basement membrane, such as those occurring in stroke, may facilitate the entry of hirudin into the brain.

### 3.2. Evidence for Brain Access

Direct evidence for brain exposure comes from radiolabeling and quantitative mass spectrometry. Technetium-99m–labeled recombinant hirudin (^99m^Tc-rH) administered intravenously to healthy mice produced low but detectable brain radioactivity, with the ID% around 0.08–0.16 in a 5–120 min time range, nearly indicating limited crossing of the intact BBB [57]. In a rabbit carotid balloon injury model receiving oral lyophilized leech extract daily (0.1 g), Ultra-Performance Liquid Chromatography-Tandem Mass Spectrometry (UPLC–MS/MS) detected tens of ng/mL of hirudin in brain tissue at 1–6 h post-dose across groups, with a brain/plasma ratio of 0.08, 0.11, 0.08 at 1, 3, 6 h respectively [58]. In addition, functional studies in stroke and hemorrhage models show stabilization of the BBB and reduction in edema and inflammation after hirudin administration, consistent with intraparenchymal activity. Together, these data suggest that hirudin can reach the brain at low levels, with access augmented under BBB-disruptive conditions. Here, we briefly list all the related outlines of the data in Table 1.

### 3.3. Putative Mechanisms of Hirudin’s BBB-Crossing

BBB-crossing peptides typically exploit receptor-mediated transcytosis (RMT) or exhibit cell-penetrating properties (CPPs) [6]. Hirudin is an anionic, 7 kDa peptide that lacks known affinity for the BBB-targeting receptor and does not exhibit canonical CPP characteristics [22,71]. Its physicochemical profile (size, charge, hydrophilicity) disfavors passive permeation across an intact endothelium.

In pathology, however, BBB integrity is compromised. Inflammatory mediators downregulate tight junction proteins (e.g., occludin, claudins, tricellulin, junction adhesion molecules), expand paracellular clefts, and alter transporter activity [72]. Under such conditions, macromolecules can enter by nonspecific, paracellular leakage driven by concentration gradients. This mechanism aligns with the low brain levels in healthy animals and higher functional effects in disease models. Notably, hirudin can suppress NLRP3 signaling and cytokine levels (TNF-α, IL-1β, IL-6, IFN-γ) and modulate claudin-5 expression [12,30], raising the possibility that brain pathology leads to more frequent paracellular access for hirudin’s entry, and hirudin then helps restore barrier function. While transcellular transcytosis cannot be ruled out, current data are most consistent with context-dependent paracellular access. Several mechanisms for brain access, and the most likely one for hirudin, are illustrated in Figure 4. Definitive mechanism-of-entry studies remain a key gap.

### 3.4. Determinants of BBB Penetration

#### 3.4.1. BBB Status in CNS Disorders

BBB permeability varies widely across diseases and over time. Ischemia–reperfusion, trauma, neuroinflammation, neurodegeneration, epilepsy, and tumors increase permeability via vasoactive agents and cytokines (e.g., glutamate, ATP, endothelin-1, nitric oxide, TNF-α, MIP-2, bradykinin, serotonin, histamine, thrombin, free radicals) [73]. In ischemic stroke, genetic and transporter changes (e.g., Slc22a8 downregulation) contribute to barrier failure [74]. External modulation, such as low-intensity pulsed ultrasound plus microbubbles (LIPU–MB), can transiently and locally open the BBB to enhance drug delivery in glioblastoma patients, though timing between sonication and infusion is critical due to rapid resealing [75].

#### 3.4.2. Molecular Properties of Hirudin

Compared with low-molecular-weight, moderately lipophilic CNS agents such as tetramethylpyrazine derivatives [76], hirudin’s higher molecular weight (~7 kDa), hydrophilicity, and anionic charge limit passive diffusion and likely contribute to rapid peripheral clearance. Electrostatic repulsion with the negatively charged endothelial surface may further reduce interaction and uptake [77]. Consequently, unmodified hirudin is unlikely to cross the intact BBB significantly.

#### 3.4.3. Administration Strategies for Hirudin

Routes of administration influence brain exposure. Intravenous dosing avoids first-pass metabolism but remains BBB-limited; intracerebral routes bypass the BBB but are invasive. Intra-arterial delivery (e.g., carotid) can provide higher local exposure; preliminary clinical experience suggests feasibility in selected cerebrovascular indications [78]. Intranasal delivery leverages olfactory and trigeminal pathways to bypass the BBB noninvasively; while high bioavailability has been reported for some agents, reproducibility can be affected by mucociliary clearance and anatomical variability [5,79]. Optimized formulations will be essential for peptide delivery via this route.

#### 3.4.4. Patient-Specific Variables

Age, sex hormones, physical activity, and comorbidities modulate BBB function [1,77]. For example, androgens and inflammatory states can alter tight junction proteins (e.g., claudin-5) and chemokines (e.g., CCL2), affecting permeability [80]. Such variability underscores the need for individualized dosing and careful monitoring in trials involving anticoagulants with potential CNS effects.

## 4. Hirudin’s Brain-Targeted Delivery System for Cerebrovascular Diseases

**Figure 5:**
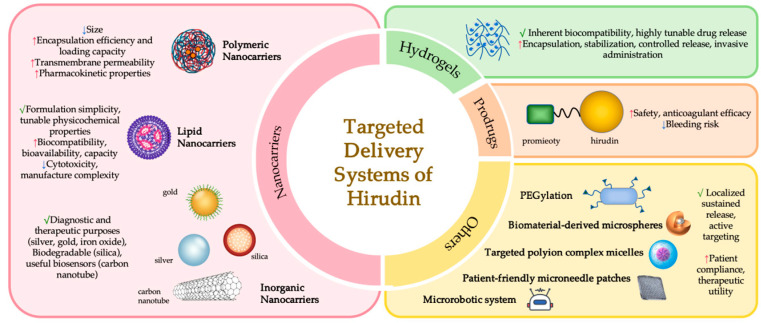
A summary of structural modifications of hirudin to enhance its targeted delivery. ↑: An enhancement effect. ↓: A reduction effect. ✓: Good characteristics.

Based on the therapeutic and BBB-penetrating analysis of hirudin discussed in previous sections, its high affinity and specificity are offset by short half-life and potential bleeding risk, resulting in a low brain/plasma ratio or ID% value [57,58]. Moreover, the clinical challenges, such as immune response and peptide degradation, remain concerns for peptide drugs, like hirudin [81,82]. Existing targeted delivery formats range from conventional preparations (e.g., lyophilized powders, capsules, injections) to advanced systems (microspheres, nanoparticles, liposomes, implants, gels, microneedles) as well as molecular engineering approaches such as PEGylation, prodrug, fusion proteins [83,84,85]. Novel strategies may offer promising solutions. Although many applications have been developed in cardiovascular or oncologic settings, the principles can translate to cerebrovascular disease, where the delivery limitations of hirudin necessitate precise targeting and controlled release. In the contents below, nanocarriers, hydrogels, prodrugs, and others are emphasized (Figure 5).

### 4.1. Nanocarrier-Based Drug Delivery Systems

A wide range of nanocarriers has been developed as delivery platforms for central nervous system diagnostics and therapeutics, with the potential to improve biodistribution, pharmacokinetics, and brain exposure [86]. Many of these systems are highly biocompatible and can be engineered to traverse the BBB, for example, through surface functionalization with targeting ligands such as peptides, thereby enhancing drug stability and brain specificity [5,87]. By enabling targeted, localized delivery, nanocarriers can increase intraparenchymal drug concentrations while reducing systemic exposure, mitigating tissue injury, supporting repair processes, and ultimately improving clinical outcomes [88].

#### 4.1.1. Encapsulation of Hirudin and Enhancement of Stability and Thrombolytic Efficacy

To overcome rapid renal clearance and proteolytic degradation, nanocarrier-based drug delivery systems have been developed to encapsulate hirudin, shielding it from enzymatic degradation and extending its plasma half-life [89,90,91]. Natural and synthetic polymer systems provide high surface area, tunable size/charge, and surface functionalization for targeting ligands, while protecting payloads from degradation and improving pharmacokinetics [92,93]. Liposomes, including solid lipid nanoparticles, nanostructured lipid carriers, and nanoemulsions, are attractive due to biocompatibility, scalable manufacturing, and capacity for both hydrophilic and lipophilic payloads [86]. Inorganic nanocarriers such as metallic (gold, silver), iron oxide, mesoporous silica, and carbon-based nanomaterials provide diagnostic and therapeutic functionality (e.g., imaging contrast, photothermal effects) and tunable loading [94].

Specifically, Jing et al. developed hirudin-loaded bovine serum albumin (BSA) nanoparticles that, upon intravenous administration, prolonged antithrombotic activity in normal rats [89]. Similarly, Mei et al. designed a core–shell complex, iNanoAOX, to encapsulate hirudin within the core, thereby protecting it from enzymatic degradation, reducing rapid systemic clearance, and demonstrating thrombolytic potential in an ischemic stroke mouse model [90]. In addition, polyester-based vehicles have achieved sustained hirudin release for up to 48 h [95]. Cationic liposomes can also enhance encapsulation efficiency and bioactivity of the anionic peptide hirudin; in preclinical models, hirudin–liposome complexes have been shown to improve bioavailability and promote favorable tissue outcomes [96,97,98]. And for inorganic nanocarrier, bivalirudin released from polydopamine-coated TiO_2_ nanotubes retained bioactivity and reduced thrombosis, improving hemocompatibility in vitro and ex vivo [99].

Simple encapsulation alone often suffers from imprecise drug release and poor targeting specificity, which can result in unintended exposure of hirudin and bleeding risk. Advanced biomimetic surface modifications of nanoparticles or functionalization of hirudin with stimulus-responsive elements can enhance the spatial and temporal control of hirudin release specifically at the thrombus site [100,101]. For example, Li et al. developed a biomimetic nanoplatform (HMPC@PM) consisting of a porphyrin-based covalent organic framework (COF)-modified melanin core coated with a platelet membrane for precise hirudin delivery. This design facilitated specific accumulation at thrombotic sites and concurrently helped inhibit thrombus recurrence [100].

#### 4.1.2. Co-Delivery of Hirudin and Adjunctive Agents Using Nanocarrier Platforms

Cardiovascular and cerebrovascular diseases involve multifactorial pathological processes that are frequently compounded by concurrent conditions such as inflammation or cancer. To achieve more integrated therapeutic outcomes, hirudin has been co-encapsulated with other bioactive agents within multifunctional nanocarriers. This integrated therapeutic action, combining anticoagulation with modulation of the disease microenvironment (e.g., through inflammation attenuation or tumor targeting), facilitates a more systematic and effective treatment regimen [27,102]. For instance, Cheng et al. developed a biomimetic thrombus-targeted nanoparticle (HMTL@PM) designed for the co-delivery of hirudin and the fibrinolytic enzyme lumbrokinase, which effectively inhibited thrombus formation and alleviated atherosclerotic inflammation [27]. Zhang et al. developed a platelet-membrane-camouflaged MnOx nanoflower system for the co-delivery of hirudin and Ag_2_S, serving as a dual theragnostic agent to synergistically treat thrombosis and cancer [102].

### 4.2. Hydrogel-Based Delivery Systems

Hydrogels are promising carriers for hirudin delivery, leveraging their inherent biocompatibility and highly tunable drug-release profiles [103,104,105,106]. The hydrophilic three-dimensional network enables effective encapsulation and stabilization of hirudin for controlled release. Furthermore, the injectability of hydrogel systems facilitates minimally invasive administration, enabling targeted delivery and prolonged retention at the site of cardiovascular and cerebrovascular lesions for spatially confined therapy. In a study by Liu et al., recombinant hirudin was incorporated into an injectable Pluronic F127 hydrogel matrix administered subcutaneously in a mouse model, resulting in sustained antithrombotic efficacy and significantly prolonged circulation time [103]. Extending this approach to brain-targeted fusion constructs is a logical next step.

### 4.3. Prodrug Strategies

The anticoagulant activity of hirudin stems from the insertion of its N-terminus into the thrombin active site. By fusing a masking peptide or protein to a prodrug, its activity can be suppressed upon administration. This prodrug circulates with enhanced stability and is selectively cleaved by thrombosis-associated enzymes such as FXa, FXIa, or thrombin, at the site of a thrombus [23,85,107,108,109,110]. This targeted activation enables precise thrombolysis and minimizes the need for repeated dosing. For example, recombinant neorudin (EPR–hirudin) is cleaved by FXa/FXIa at thrombus sites to release active hirudin (HV2), demonstrating low toxicity in primates and completing phase I evaluation [21,111,112]. In addition, Zhu et al. engineered a hirudin-based prodrug by conjugating it with two functional domains to enhance circulation time and targeting capabilities. This construct exhibited high affinity for both human serum albumin and procoagulant platelets in vitro. It facilitated targeted accumulation and reduced occlusive thrombus formation in a rat carotid artery injury model [107].

### 4.4. Other Delivery Systems

A range of innovative delivery platforms, including PEGylation, biomaterial-derived microspheres, targeted polyion complex micelles, patient-friendly microneedle patches, and microrobotic systems, have also been developed to improve the delivery of hirudin and address various pharmacokinetic and clinical challenges [91,113,114,115].

PEGylation: PEGylation involves the covalent attachment of polyethylene glycol, which increases hydrodynamic size, reduces proteolysis and immunogenicity, and prolongs circulation time [115]. In addition, combining PEGylation with a targeted ligand can further enhance delivery efficiency. Rational cysteine substitution enabled site-specific PEGylation of hirudin variant 3 with improved pharmacokinetics [116], and a phase II study compared PEG–hirudin (SPP200) with heparin in hemodialysis [117].

Biomaterial-derived microspheres: Kudo et al. developed a collagen microsphere system for sustained delivery of hirudin, using calf dermis collagen as the carrier. After intrathecal administration in canine models, the system maintained effective drug concentrations in cerebrospinal fluid, significantly alleviating cerebral vasospasm and suppressing inflammatory cell infiltration [113].

Polymer micelles: In addition to these platforms, biofilm-coated nanocarriers and nucleic acid self-assemblies offer further options for controlled release and targeted delivery in CNS disease [93]. Wang et al. developed two polyion complex micelle systems based on chitosan derivatives that specifically bind to platelets, thereby extending the systemic retention of hirudin and stabilizing its plasma concentration [91]. The resulting enhanced anticoagulant and antiplatelet aggregation effects may facilitate targeted delivery and prolonged circulation of hirudin.

Transdermal microneedle patches: Microneedles are being explored as a patient-friendly alternative for hirudin delivery. Men et al. fabricated a soluble microneedle patch using PVP and PVA as the matrix and loaded with hirudin, which promoted complete wound healing shortly after administration and caused minimal irritation compared to conventional injections [114]. Incorporation into microneedles enabled simplified, long-acting antithrombotic therapy with on-demand recovery [23]. Additionally, Wu et al. developed a 3D-printed PLGA microneedle array for transdermal delivery of recombinant hirudin, demonstrating both thrombosis prevention in a thromboembolic disease model and the feasibility of long-term patient self-administration [104].

Microrobotic system: Cong et al. developed a biomimetic delivery system by encapsulating a staphylokinase-hirudin fusion protein (SFH) within Escherichia coli BL21-derived outer membrane vesicles (OMVs), which were subsequently engineered into two microrobotic platforms. These systems demonstrated potent thrombolytic activity in vitro and in vivo with reduced risk of bleeding [108].

## 5. Proposal of Hirudin in Its Therapeutic Potential for Other Brain Diseases

Hirudin’s anticoagulant and anti-inflammatory actions make it an appealing partner in rational combinations that address multifactorial CNS pathology. In peripheral neuropathy models, combining hirudin with cinnamaldehyde yielded additive cytoprotection via Nrf2/HO-1 upregulation and nuclear factor kappa-B (NF-κB) suppression [118], illustrating complementary mechanisms that could translate to the CNS. With appropriate targeting and controlled activation, hirudin can be engineered as a “therapeutic–carrier” that both modulates brain pathology and helps co-deliver other agents. Below, we highlight applications in stroke, neurodegeneration, psychiatric disorders, and brain tumors.

### 5.1. Stroke Treatment with Modified Hirudin

Clinical translation is challenged by short half-life, bleeding risk, and species differences [84]. Nanocarriers that are home to thrombi and ischemic tissue can raise local drug levels within the therapeutic window and minimize systemic exposure. Hirudin-loaded biomimetic nanoparticles that accumulate at thrombi and modulate inflammation and cholesterol efflux in atherosclerosis [27] could be adapted for cerebrovascular use by integrating BBB-targeting ligands. Platform approaches that extend rtPA half-life and reduce hemorrhage (e.g., discoidal particles with magnetic guidance) [119] could co-deliver hirudin to combine thrombolysis with sustained thrombin inhibition. Localized release (e.g., stent coatings eluting PEG–hirudin and vasodilators) has reduced restenosis in large-animal models [120] and may support peri-procedural anticoagulation in neuro-interventions.

### 5.2. Neurodegenerative Diseases Treatment with Hirudin

Alzheimer’s disease (AD): Vascular dysfunction contributes to AD pathogenesis, and improving cerebral perfusion may aid management [121]. A 20-week pilot study reported that hirudin plus donepezil improved cognition more than donepezil alone in mild-to-moderate AD, with acceptable safety [121]. Nanotechnology offers routes to address core pathologies: reactive oxygen species (ROS)-responsive micelles that reprogram microglia and inhibit Aβ improved cognition in transgenic models [122], and cationic liposomes with dual BBB/microglia targeting enhanced outcomes in late-stage mice [123]. Molecular engineering of multi-target prodrugs (e.g., donepezil–memantine–Aβ binders) improved efficacy and reduced side effects in AD models [124], while BBB-shuttling fusion biologics (e.g., single-domain antibody FC5 fused to Aβ-oligomer binders) reduced brain Aβ after systemic dosing [125]. Within such frameworks, hirudin could be paired with Aβ/tau-directed agents to provide vascular and anti-inflammatory support as a therapeutic agent while potentially aiding delivery as a delivery agent when BBB integrity is compromised.

Parkinson’s disease (PD): PD features dopaminergic neuron loss, α-synuclein aggregation, neuroinflammation, and BBB alterations [126,127,128]. Suppressing NLRP3 signaling can reduce α-synuclein pathology and neurodegeneration [129]. Targeted delivery via transferrin receptor–decorated liposomes increased brain uptake of an anti-α-synuclein antibody sevenfold and improved motor and cognitive function in mice [130]. Analogous systems that co-deliver hirudin (to dampen thrombin/PAR-driven inflammation as a therapeutic) with anti-synuclein therapeutics warrant exploration. Given a likely intact BBB in early PD, brain targeting ligands or transient BBB modulation may be required; unmodified hirudin alone is unlikely to achieve substantial CNS exposure in such contexts.

### 5.3. Psychiatric Disorders Therapies with Hirudin

Over a billion people are affected by mental disorders globally, with anxiety and depression most prevalent [131]. BBB restriction, peripheral metabolism, and physicochemical limitations reduce central exposure of many psychotropics [132,133]. Modifying drugs with novel delivery platforms could significantly enhance the therapeutic outcome. For example, nanocarriers can protect labile drugs, improve solubility, and enhance brain delivery [134]. One study found that amitriptyline-loaded copolymeric nanoparticles improved antidepressant and anxiolytic behavior in animal models [135]. Also, fusion strategies that modulate synaptic machinery implicated in antidepressant action (e.g., SNARE complex dynamics) have mechanistic appeal [136]. In this space, hirudin’s role would likely be adjunctive and engineered, e.g., incorporated into targeted carriers or prodrugs that exploit disease-related BBB changes, since its intrinsic BBB penetration is limited when the barrier is intact. There is also evidence that the combined use of hirudin and donepezil improves the psychiatric outcome in AD patients [121]. Thus, hirudin may serve both as a therapeutic agent for regulating the syndromes and as a delivery molecule for other drugs. Anti-inflammatory actions might be relevant in disorders with prominent neuroinflammation (e.g., schizophrenia); however, further assessment of bleeding risk is essential when hirudin is combined with common antidepressants, such as selective serotonin reuptake inhibitors (SSRIs), serotonin-norepinephrine reuptake inhibitors (SNRIs), and tricyclic antidepressants (TCAs), which can increase bleeding when co-administered with anticoagulants [137].

### 5.4. Brain Tumor Treatment with Hirudin

Gliomas present severe delivery challenges due to the BBB and the heterogeneous blood–brain tumor barrier (BBTB). Passive accumulation via the enhanced permeability and retention effect is inconsistent across tumor grades and regions; high-grade glioblastoma can exhibit larger vascular fenestrations, but coverage is patchy [138,139,140]. Active targeting via receptors such as transferrin receptor, LRP1/2/8, insulin receptor, or nicotinic acetylcholine receptors can improve intratumoral delivery [139]. PEGylation extends nanoparticle circulation and, combined with RMT ligands, can enhance BBB transit and antitumor efficacy [141]. Hirudin could be integrated as a co-therapeutic (e.g., anti-thrombotic/anti-inflammatory) or as part of multi-drug payloads. Intrinsically, hirudin has been reported to inhibit glioma growth and modulate autophagy via mTOR signaling in xenografts [59]. Given the high incidence of perioperative and disease-related thrombosis in brain tumor patients [142], dual antitumor and antithrombotic strategies are clinically attractive, provided bleeding is carefully controlled.

## 6. Conclusions

In summary, hirudin has progressed from a natural anticoagulant to a candidate CNS therapeutic with context-dependent brain access and neuroprotective activity in stroke. Its ability to reach the brain appears limited under physiological conditions but increases when the BBB is disrupted, consistent with paracellular leakage. This property, combined with potent thrombin inhibition and anti-inflammatory effects, positions hirudin as both a therapeutic and a potential component of brain-delivery platforms. Advances in nanocarriers, hydrogels, prodrugs, and others have improved stability, targeting, and safety in preclinical models. Building on the evidence and strategies discussed throughout this review, several promising directions for future research and development emerge.

1. Rational carrier design: Stimuli-responsive (e.g., ROS/pH) systems capable of high on-target release in diseased microenvironments, combined with BBB-targeting ligands (e.g., transferrin, lactoferrin) to harness carrier-mediated transport (CMT) or RMT, represent a compelling avenue [143]. Advances in manufacturing control, including predictive models of nanoparticle growth and release, will be critical for clinical translation [144]. Ongoing formulation efforts may indirectly improve the immunogenicity and peptide degradation profiles of hirudin-based therapeutics.
2. Multimodal therapy: Systematic evaluation of hirudin in combination with thrombolytics, neuroprotectants, anti-amyloid/tau or anti-synuclein agents, and immunomodulators warrants further investigation. Where appropriate, transient BBB opening (e.g., ultrasound) or alternative routes (e.g., intranasal) could be leveraged to enhance delivery [75,145].
3. Quantitative monitoring: Affinity capture coupled with LC–MS/MS platforms shows promise for characterizing CNS pharmacokinetics and could be adapted for brain matrices to support rigorous pharmacodynamic assessments [146]. Establishing the causal relationship between adequate brain exposure, target thrombin inhibition, and the resulting therapeutic outcome is crucial.
4. Translational models: Beyond rodent studies, validation in nonhuman primate stroke models and human-relevant systems such as BBB-on-chip or brain organoids will be essential to bridge the translational gap [147,148].
5. Indication expansion: Beyond stroke, hirudin-based platforms could be explored in other CNS disorders, including Huntington’s disease and amyotrophic lateral sclerosis, where rational peptide designs targeting pathogenic protein aggregation may be applicable [149].
6. Regulatory and chemistry, manufacturing, and controls (CMC) readiness: Early definition of critical quality attributes for engineered hirudin products, along with coordinated process development and supply chain planning, will help avoid delays due to CMC immaturity [150]. Established platforms such as CPPs, nanocarriers, and receptor-mediated transport systems should also be used as benchmarks.

Collectively, with continued attention to quantitative pharmacology, precision targeting, and manufacturability, hirudin-based platforms hold promise to contribute meaningfully to a new generation of CNS therapies.

## Figures and Tables

**Table 1:** Research evidence of hirudin’s BBB permeability.

Diseases and Models	Dose and Administration	Results	Ref
Ischemic stroke and OGD/R model in brain microvascular endothelial cells (BMECs) of rat	Varying concentrations of hirudin (0, 10, 20, 40, 80, and 160 µg/mL) for 24 h	Hirudin significantly improved BMEC survival and enhanced both cell migration and tube formation	[30]
Cerebral ischemia–reperfusion injury (CIRI) and OGD/R model in HT22 cells	Varying concentrations of hirudin (5 μM, 10 μM, and 20 μM) for 24 h	Hirudin enhanced neuronal viability and reduced apoptosis in OGD/R-stimulated cells	[13]
Acute ischemic stroke and ODG/R model in BV-2 microglia cells	Varying concentrations of hirudin (ranging 1.25–20 μM) for 24 h with the exposure to 4 h OGD	Hirudin protected the viability and death of BV-2 microglia cells against OGD/R	[12]
Glioma and 3 glioma cell lines U251, LN229 and U87MG	Varying concentrations of hirudin (1, 2, 6, 8 U/mL) or times	Hirudin dose- and time-dependently inhibited glioma invasion, migration and proliferation	[59]
Apoptotic effect of thrombin, and BMEC model of cultured rat	100 μM/L hirudin for 30 min	Hirudin has a definite blocking effect on the damage caused by thrombin to BMECs	[60]
Ischemic stroke and MCAO/R rat model	Intraperitoneal injection of hirudin (40 mg/kg), initiated 24 h after surgery and administered once daily for 7 consecutive days	Hirudin reduced the modified neurological severity (mNSS) score, alleviated pathological damage, decreased infarction volume, and increased the expression of key angiogenic factors	[31]
CIRI and MCAO mice model	Intracerebroventricular injection of hirudin (40 mg/kg) and administered once daily for 14 consecutive days	Hirudin significantly improved neurological function and reduced cerebral edema and infarct size in the MCAO model	[13]
Acute ischemic stroke and MCAO/R mice model	Oral administration of hirudin (10, 20, and 40 mg/kg) via a gavage	Hirudin markedly constrained cerebral infarct area in a dose-dependent manner, and significantly improved locomotor disability at 40 mg/kg dose	[12]
Cerebral ischemia and MCAO rat model	Intracerebroventricular injection of hirudin (10 U) 30 min after the MCAO induction, and administered on days 1, 3, and 7 following MCAO	Hirudin improved cognitive and motor deficits post-ischemia, reduced brain infarction, neurological damage and oxidative stress, also enhanced neurogenesis in ischemic rats	[61]
Acute ischemic stroke and MCAO rat model	Intraperitoneal injection of hirudin (2 mg/kg) 2 h after MCAO and administered once daily for 7 consecutive days	Hirudin protected the BBB function of the brain tissue in the infarcted area	[62]
Intracerebral hemorrhage (ICH) and ICH rat model	Original hematoma location injection of hirudin (10 U in 15 μL saline) at 5 min and 24 h after ICH	The early application of hirudin after ICH could significantly reduce microglia and neutrocyte expression	[63]
ICH and ICH mice model	Intraperitoneal injection of hirudin (60 ATU/kg) for 21 consecutive days starting at day 7 post ICH	Hirudin reduced leukocyte accumulation in the brain and shifted microglia toward an anti-inflammatory phenotype	[64]
ICH and ICH rat model	Caudate nucleus injection of hirudin (10 U in 3 μL saline) 5 min after ICH	Hirudin reduced both brain water content and BBB permeability	[65]
ICH and ICH rat model	Caudate nucleus injection of hirudin (15 U in 3 μL saline) 5 min after ICH	Hirudin alleviated both brain edema and BBB permeability	[66]
ICH and ICH rat model	Caudate nucleus injection of hirudin (15 U in 3 μL saline) 5 min after ICH	Hirudin relieved brain edema and permeability of BBB after ICH	[67]
ICH and ICH rat model	Caudate nucleus injection of hirudin (15 U in 3 μL saline) 5 min after ICH	Hirudin lowered the BBB permeability	[68]
Acute ICH and 120 acute ICH patients	Intravenous drip of hirudin injection (6 mL) for 21 consecutive days	Hirudin reduced intracranial pressure, promoted the absorption of cerebral hemorrhage edema, shrank the low-density areas around the hematoma, and facilitated the recovery of the patient’s neurological function	[69]
SAH and SAH rat model	Lateral ventricle injection of hirudin (10 U) at 30 min, 24 h, and 48 h post-SAH	Hirudin significantly ameliorated neurological scores and attenuated brain edema, BBB permeability, inflammatory response, microglia activation, and pyroptosis	[30]
Glioma and Cell-derived xenograft (CDX) nude mice	Intraperitoneal injection of hirudin (2 U, 4 U/mL), and administered once daily for 21 consecutive days	Hirudin inhibits the tumour growth via inducing autophagy in CDX nude mice	[59]
Apoptosis of cerebellar granule neurons (CGNs) and CGN rat model	Intravenous drip of hirudin (2, 2.5, 3.34, 5 and 10 U/mL)	The apoptosis rate changed slightly between neurons with different hirudin concentrations, but the higher concentration, the lower the apoptosis rate	[70]

## Data Availability

No new data were created. Data sharing is not applicable to this article

## Abbreviations

The following abbreviations are used in this manuscript:

AD	Alzheimer’s disease
AMT	Adsorption-mediated transcytosis
BBB	Blood–brain barrier
BMEC	Brain microvascular endothelial cell
CDX	Cell-derived xenograft
CGN	Cerebellar granule neuron
CIRI	Cerebral ischemia–reperfusion injury
CMC	Chemistry, manufacturing, and controls
CNS	Central nervous system
CPP	Cell-penetrating property
Cys	Cysteine
Da	Dalton
DVT	Deep vein thrombosis
EVT	Endovascular therapy
INR	International normalized ratio
LMWH	Low-molecular-weight heparin
MCAO/R	Middle cerebral artery occlusion/reperfusion
mNSS	Modified neurological severity
NF-κB	Nuclear factor kappa-B
NLRP3	NOD-like receptor protein 3
NVU	Neurovascular unit
OGD/R	Oxygen-glucose deprivation/reperfusion
PD	Parkinson’s disease
RMT	Receptor-mediated transcytosis
ROS	Reactive oxygen species
rtPA	Recovery via thrombolysis
SAH	Subarachnoid hemorrhage
SNRIs	Serotonin-norepinephrine reuptake inhibitors
SSRIs	Selective serotonin reuptake inhibitors
TCAs	Tricyclic antidepressants
TJ	Tight junction
UPLC-MS/MS	Ultra-Performance Liquid Chromatography-Tandem Mass Spectrometry
